# Mechanistic Study of the Formation of Multicomponent Oxide Porous Microspheres (MICROSCAFS^®^) by Cryo-Scanning Electron Microscopy

**DOI:** 10.3390/gels9090704

**Published:** 2023-09-01

**Authors:** Mário Vale, Ana C. Marques

**Affiliations:** CERENA, DEQ, Instituto Superior Técnico, Universidade de Lisboa, Av. Rovisco Pais, 1049-001 Lisboa, Portugal

**Keywords:** cryo-SEM, porous microspheres, MICROSCAFS, silica, titania, sol–gel, phase separation, macroporosity

## Abstract

Multicomponent oxide microspheres with interconnected macroporosity (MICROSCAFS^®^) are new materials with great potential as support materials for photocatalysis, optimized for real life applications and for other uses that are still being explored. They are obtained from an adapted sol–gel process combined with phase separation phenomena that occur within the water droplets of an emulsion. We present here a methodology based on cryogenic scanning electron microscopy (cryo-SEM) that allows, with minimal specimen preparation, the direct and in situ visualization of ‘wet’ alkoxide-derived microstructures, for the mechanistic study of the complex process of MICROSCAFS^®^ generation. It is simultaneously combined with energy dispersive X-ray spectroscopy (EDS) to visualize phase separation phenomena and study the chemical elemental composition at specific regions of the sample and reaction times.

## 1. Introduction

Sol–gel-derived silica, silica–titania (ST) and silica–titania–hafnia (STH) microspheres with controlled and tailored interconnected macroporosity (MICROSCAFS^®^) have been achieved by microemulsion techniques combined with polymerization induced phase separation, without the need for the employment of a phase separation additive [1,2,3]. The microspheres are templated by the water droplets of the water-in-oil (W/O) emulsion. The inherent gelation capability of the involved silanes, with particular emphasis on 3-(glycidyloxypropyl)trimethoxysilane (GPTMS), as well as the employment of the titanium (Ti) and hafnium (Hf) precursors and the control of pH, temperature, and water content, allow the good mastering of the MICROSCAFS^®^ production technique, enabling a fine-tuning of their pore size morphology and reproducibility of their characteristics [3]. These new products have been evaluated for solar-driven photocatalysis as support materials in which photocatalytic nanoparticles are chemically bound and are of potential interest for various applications in energy, the biomedical field, and chromatography, among others.

However, the main phenomena happening inside the water droplets of the microemulsion, critical for the interconnected macroporosity generation, such as the way macromolecules assemble within the colloidal solution (sol) and the phase separation evolves, are still not completely understood. This study is critical to understand how the MICROSCAFS^®^, namely their characteristic porosity, are generated and, therefore, it is important to fine-tune their pore morphology and assure reproducibility of the process. Herein, we show how cryo-scanning electron microscopy (Cryo-SEM) is a viable technique to provide us the answers for the MICROSCAFS^®^ formation inside the emulsion water droplets. 

The cryo-SEM microscope is composed of a SEM base unit annexed to a cold stage and to a cryo-chamber where the coating, cutting, and etching (ice sublimation) are performed. This system is cooled by a single nitrogen tank and is operated constantly under vacuum. The first stage of the process is the preparation of the sample. It can be performed outside or inside the cryo-SEM, depending on the available equipment. If performed outside, a liquid- or moisture-containing sample is rapidly frozen by plunging into sub-cooled nitrogen, often called "slush nitrogen”. Then, the specimen is transferred to the cryo-chamber stage with the help of an airlock system and processed. The sample is cut with a cold knife apparatus for the analysis of its internal structure. Then, the ice on the surface is removed by sublimation using a heater in a controlled manner. This process is called etching and is crucial because the ice would obscure the structure of interest and produce charging artifacts which can render the visualization of the specimen nearly impossible. Finally, it can be coated with a conducting layer, normally composed of 15 nm of Au-Pd, before being transferred to the cold microscope stage.

In recent years, studies involving cryo-SEM applied to emulsions have been reported, comprising mainly the study of Pickering emulsions, mostly in the food science field [4,5,6,7,8,9,10,11,12,13]. Some of these works are focused on the development of high internal phase emulsions (HIPEs) [5,8,12], which are highly concentrated gelled emulsions with an internal (dispersed) phase volume fraction exceeding 0.74 [14], and some of them involve metallic–organic frameworks (MOFs) [9,11] or silica nanoparticles [13] as Pickering stabilizers. Cryo-SEM has also been employed to study the stability of emulsions in food science [15,16,17,18] to analyze the fat crystallization properties inside the oil droplets of an oil-in-water (O/W) emulsion [19] as a tool for the development of natural sorbents for the removal of toluene from water [20] and to study a delivery system of lipophilic bioactive compounds [21]. 

Regarding the sol–gel research field, cryo-SEM has been employed to observe the gelling point of a chelated aluminum alkoxide system [22]. A wavy matrix characteristic of the formation of polymeric alumina was spotted after the hydrolysis of the precursors in a very reproducible way. This technique has also been used to characterize the structure and morphology of silica/polysaccharide nanocomposites for the development of novel materials with tailored properties [23] and for the structure elucidation of silica-based microcapsules containing drugs for topical applications [24]. In this latter case, Erlich et al. [24] studied, by cryo-SEM, two different types of microcapsules that contained active pharmaceutical ingredients used for the treatment of skin diseases, namely benzoyl peroxide (BPO) and tretinoin (ATRA). These two APIs, when used together in the same formulation, provide a very effective topical anti-acne solution due to their complementary mechanism of action. However, ATRA needs to be encapsulated to be protected against oxidative decomposition by BPO, while BPO, a strong oxidizer, needs to be encapsulated to prevent skin irritations. Encapsulation by a porous silica shell provides a barrier between the drugs and the skin and a slow release required for a correct treatment. The encapsulation of each API involved two different manufacturing processes based on the sol–gel technology employing different silica precursors according to their specific chemical properties. The authors observed both microcapsules through cryo-SEM using a secondary electron detector and a backscattered electron detector and compared them with the corresponding non-encapsulated APIs. The samples were frozen and kept in their fully hydrated state, assuring in this sense that their characteristic morphology was intact. The effectiveness of the encapsulation process was proved by this technique and the authors proposed the use of this methodology for the structural analysis of a wide range of aqueous and non-aqueous suspensions of nano and microparticles. It should be stressed, however, that this study only involved microcapsule samples in their last stage of production and not at intermediate times of their synthesis, i.e., it does not consider a mechanistic study of the capsule formation.

The target of the work reported herein is a first-time mechanistic study of the microsphere (MICROSCAFS^®^) formation process, i.e., of the sol–gel and phase separation phenomena in their first stages, when the microspheres are still in their embryonic stage. For such purpose, cryo-SEM was employed, i.e., aliquots were taken from the reaction batch at specific times during the synthesis of the MICROSCAFS^®^, cryogenized, and analyzed, either by the secondary electrons (SE) mode, which topographically offers information of the fractured surfaces, and the backscattered electrons (BE) mode, which provides contrast between elements of different atomic numbers. This procedure was at the same time combined with energy-dispersive X-ray spectroscopy (EDS) to provide answers through the observation of the phase separation phenomenon that occurs inside the water droplets of the emulsion and leads to the characteristic interconnected macroporosity, and the study of the chemical elemental composition at specific regions of the samples and reaction times.

## 2. Results and Discussion

**Figure 1:**
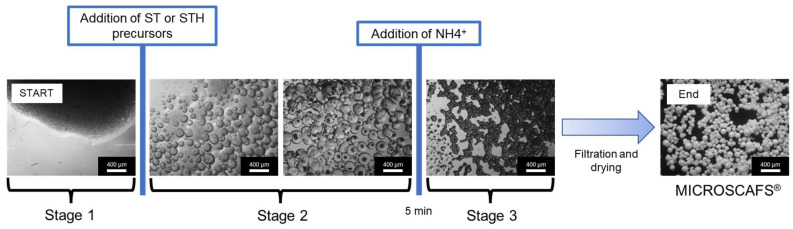
Different stages of the MICROSCAFS^®^ synthesis process, observed by optical microscopy. Scale bar = 400 µm.

We divide the ST and STH MICROSCAFS^®^ synthesis into three stages, represented by the optical microscopy images of Figure 1. Stage 1 consists of the simultaneous preparation of the emulsion and of the precursors colloidal solutions (sol), Stage 2 (embryonic stage) starts with the incorporation of the sol into the previously prepared emulsion, and Stage 3 starts after the addition of the ammonia (NH_4_^+^) solution and ends when the MICROSCAFS^®^, in the gel form, are formed. The dried MICROSCAFS^®^ were observed by SEM. Samples for cryo-SEM observation were prepared by taking an aliquot at these three different stages (Figure 1) and rapidly freezing it through immersion in liquid nitrogen, which was also complemented with EDS to study the chemical elemental composition at specific reaction times and locations in the samples (Table 1).

**Figure 2:**
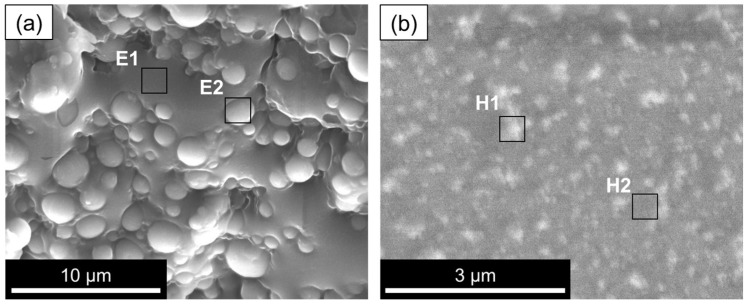
Cryo-SEM images of (**a**) W/O emulsion and (**b**) ST pre-hydrolysate (Stage 1).

The SE detector micrograph shows the topography of the W/O emulsion, the first step of the microsphere synthesis process. As expected, the W/O emulsion displays water droplets rich in oxygen (ca. 90 at%) within the oil phase (decalin) of the emulsion. This latter one is rich in carbon (ca. 93 at%) (Figure 2a, regions E2 and E1, respectively).

In parallel to the emulsion, the pre-hydrolysate, i.e., a sol made from the metal precursors, such as TEOS, GPTMS, and TiPOT, was also observed by cryo-SEM. Figure 2b, in the BE mode, exhibits a silica–titania sol before being added to the emulsion, displaying oligomeric clusters represented by the whitish regions. The greyish regions (H2) also display the presence of Si and Ti, slightly less, however, than the whitish regions (H1), as well as some Cl due to the hydrolysis catalyst (HCl) employed in the synthesis. Also, in Figure 2b, the content of Ti is slightly higher compared to that of Si (Ti/(Si + Ti)) = 19% in region H1 versus 16% for the darker region H2.

**Figure 3:**
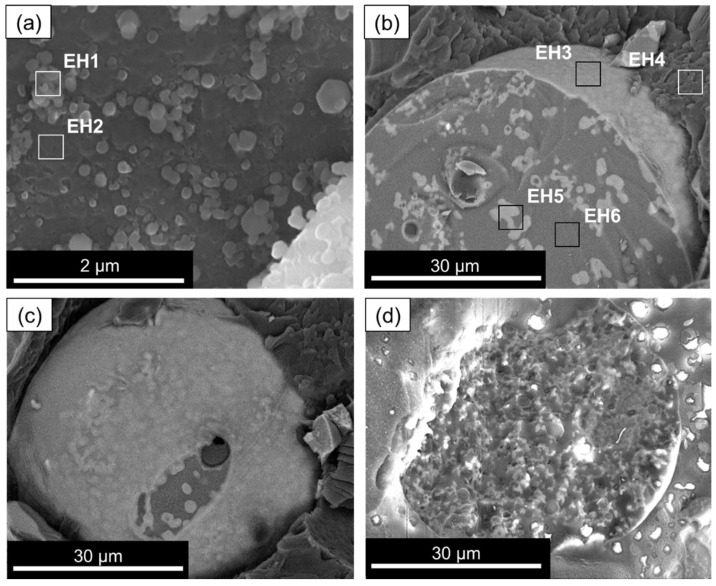
Cryo-SEM images of (**a**) ST oligomer clusters in the water phase, (**b**) STH MICROSCAFS^®^ (cross-section) in the embryonic stage (BE mode), (**c**) STH MICROSCAFS^®^ in the embryonic stage (BE mode), (**d**) ST MICROSCAFS^®^ (cross-section) in the embryonic stage (SE mode) (Stage 2).

The addition of the pre-hydrolysate to the emulsion leads to some destabilization, namely droplet (aqueous phase) coalescence, which acts as a template for the MICROSCAFS^®^ round shape. A sample of the aqueous phase of the emulsion, collected in the beginning of Stage 2, was observed by cryo-SEM in the SE mode. It is essentially composed of the added sol but a bit more diluted (lesser content of carbon atoms in both regions, EH1 and EH2) than the pre-hydrolysate, which confirms that the pre-hydrolysate (silanols, Si-OH) migrates to the water droplets of the emulsion due to its affinity with water. Also, the clusters (EH1) are found to increase in size, and they become richer in Si and Ti atoms, and poorer in C atoms, revealing the progress of hydrolysis and condensation reactions. As time proceeds, polymerization of silicate species occurs: first, it is the polymerization (condensation reactions) of monomers and oligomers containing Si-OH and Ti-OH groups to form particles and clusters (observed in Figure 3a) with Si-O-Si and Si-O-Ti bonds. This releases by-products such as alcohol and water. Second, further growth of the agglomerates and clusters occurs, as well as their linking into chains, and third, formation of networks that extend throughout the liquid medium proceeds, resulting in a gel. Since TiPOT is more reactive than the Si precursors employed, the polymerized clusters (condensation products, region EH1), which probably have higher molecular weight than the starting oligomers, are found to be richer in Ti atoms than the neighboring region EH2. Region EH1 of Figure 3a, shows an agglomerate of clusters exhibiting a Ti/(Si + Ti) = 26%, while region EH2 exhibits Ti/(Si + Ti) = 14%.

**Figure 4:**
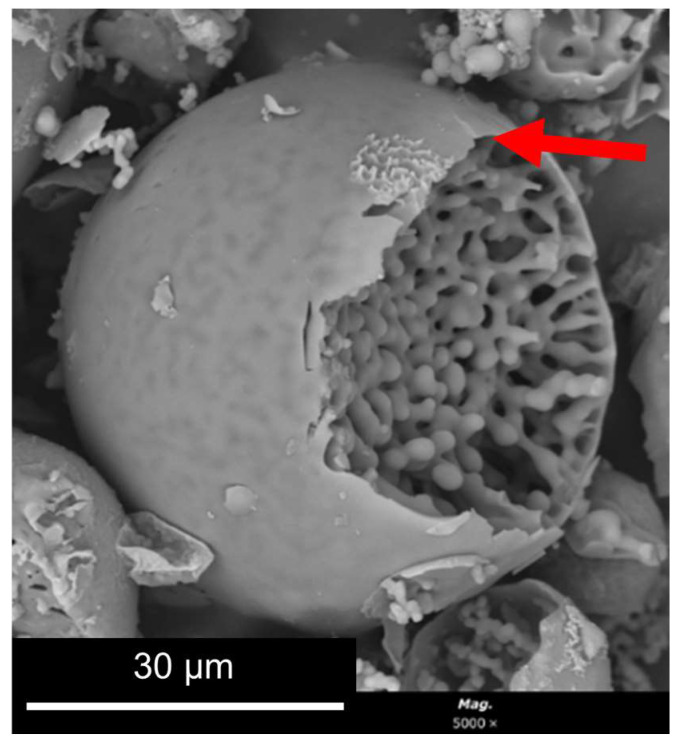
Example of a MICROSCAFS^®^ final morphology.

Figure 3b–d show microspheres in their embryonic stage taken at the end of Stage 2. The oligomeric clusters, present in the aqueous phase of the emulsion, are shown to accumulate at the water/oil interfaces (Figure 3b, region EH3, displaying the following relative atomic %: 80% Si, 6.6% Ti, 13.5% Hf), possibly to reduce the surface energy, explaining the presence of the thin layer that wraps the microspheres. It should be noted that this thin layer remains and is visible at the dried MICROSCAFS^®^ (Figure 4, arrow). This is followed by the formation of polymerized skeleton domains by condensation reactions (Figure 3b, region EH5, displaying the following atomic % for an STH composition: 73.6% Si, 17% Ti, 9.2% Hf). The regions around these skeleton domains (Figure 3b, region EH6) are richer in oxygen, which reveals the presence of an aqueous phase around them, but also containing Si, Ti, and Hf atoms, probably oligomers or polymerized fractions of lower molecular weight (Mw) compared to that of the skeletons (Figure 3b, region EH5). This reveals the early occurrence of phase separation between the water-rich phase and the polymerized fractions. With time, these oligomers or polymerized fractions of lower Mw join the formed skeletons, increasing the polymerized skeleton fraction within the microsphere, which grow by gelation, concomitantly with phase separation, until the microspheres attain their final morphology (Figure 4). 

Region EH4 shows that the zone involving the embryonic microspheres is richer in carbon, like region E1 in Figure 2a, confirming that the microspheres migrate to the oil phase once they start to form, surrounded by decalin (oil phase). In some cases, chlorine atoms are detected, which originate from the hafnium precursor or eventually from the pre-hydrolysis catalyst employed, HCl.

**Figure 5:**
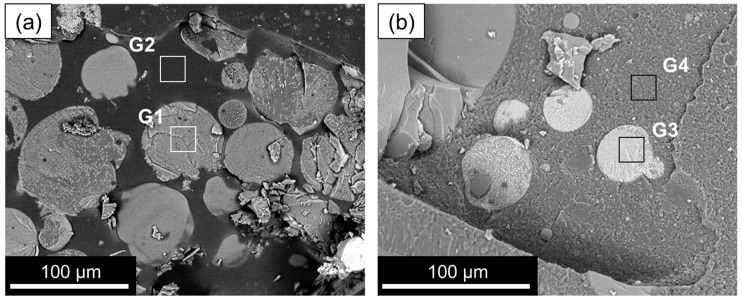
Cryo-SEM images of (**a**) ST and (**b**) STH MICROSCAFS^®^ after the addition of ammonia (Stage 3).

The addition of ammonia is found to catalyze the gelation process. Figure 5 shows wet gel MICROSCAFS^®^ particles, after ammonia addition (Stage 3) for the ST composition (region G1, displaying particles with a relative atomic % of 76% Si and 23% Ti) and STH (region G3, displaying particles with a relative atomic percentage of 76.5% Si, 16% Ti and 6.5% Hf). The content of oxygen is relatively high (ca. 72%) in regions G1 and G3, which reveals that water from the initial aqueous phase and resulting from polycondensation is entrapped inside the microspheres, namely in the water-rich phase, formed by phase separation. 

Again, it is shown that the formed microspheres are in the oil phase (Figure 5, regions G2 and G4) since these zones are rich in carbon and sparse in oxygen, similar to the oil phase of the emulsion (Figure 2, region E1).

The water entrapped inside the microspheres is removed by drying/heat treatment after the microspheres are collected, filtered, and washed at the final stage of the MICROSCAFS^®^ synthesis procedure.

**Figure 6:**
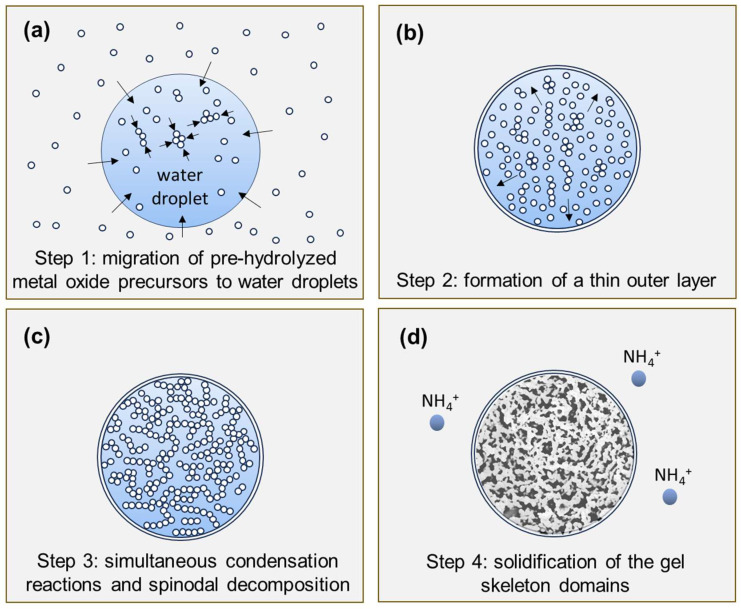
Formation mechanism of the multioxide MICROSCAFS^®^.

With the help of the cryo-SEM characterization, we propose the following mechanism for the formation of the multioxide MICROSCAFS^®^ (Figure 6):

Step 1 the pre-hydrolyzed metal oxide precursors (sol) migrate to the water phase agglomerated droplets of the emulsion with the formation of oligomeric clusters (Figure 6a).
Step 2 accumulation of the oligomers at the water–oil interface, either by migration or kinetically trapping, and formation of a nanometric outer layer prior to the development of the internal interconnected gel skeleton, isolating the internal water/oligomer system from the outside (oil phase) and preventing particle coalescence (Figure 6b).
Step 3 condensation reactions lead to the formation of Si-O-Si, Si-O-Ti bonds at the expense of Si-OH and Ti-OH, with the formation of Si- and Ti-rich skeleton domains and their separation from the water phase inside the droplets (Figure 6c), typically by spinodal decomposition.
Step 4 concomitant phase separation and gelation with the system quickly maturing after the addition of the ammonia catalyst, producing the final MICROSCAFS^®^ with interconnected macroporosity (Figure 6d). The typical mesoporosity observed in the MICROSCAFS^®^ arises mainly from the binding of oligomeric clusters, forming the skeleton domains, while interconnected macroporosity is due to the phase separation process by spinodal decomposition. The size of the oligomeric clusters affects the size of the mesopores and can be influenced by the pH of the reaction medium, temperature, or the use of specific surfactants, such as the amphiphilic Pluronic^®^ P123. Post-synthesis procedures, based on solvent exchange, consist of another alternative.

We have demonstrated in previous works that by changing the synthesis parameters, namely precursor content, temperature, pH, etc., we can obtain either smaller or larger interconnected porosity and different particle sizes [2,3]. This is in line with the formation mechanism previously shown, where kinetics (hydrolysis and condensation reactions) and thermodynamics phenomena simultaneously occur. Also, the formation of a shell outside the system provides a more controlled, stable and favorable environment inside the water droplets for the thermodynamics phenomenon, i.e., phase separation by spinodal decomposition, to occur. Phase separation onset and gelation times are therefore more controlled, allowing the fine tuning of the final porosity and particle sizes by changing the synthesis parameters.

## 3. Conclusions

Cryo-SEM complemented by EDS, SEM and optical microscopy proved to be an effective way to study the sol–gel chemistry of SiO_2_-TiO_2_ and SiO_2_-TiO_2_-HfO_2_ compositions at their first stages, as well as the phase separation phenomenon which occurs in parallel, for the generation of multioxide microspheres with interconnected macroporosity. We were able to observe highly magnified images of the emulsion that serves as a template for the MICROSCAFS^®^ generation, identify the water and oil phases by EDS and understand the way the emulsion evolves during this synthesis. Oligomers are shown to homogeneously form within the aqueous phase (agglomerated droplets) of the emulsion and accumulate at the water–oil interface, explaining the presence of the thin layer that typically wraps the MICROSCAFS^®^. This is followed by formation and growth by gelation of skeleton domains, concomitantly to phase separation, until the microspheres achieve their final interconnected macroporous morphology. Cryo-SEM was essential to gain the first insight into the generation process of the MICROSCAFS^®^, elucidating their formation mechanism, proving to be critical for the optimization and development of advanced materials with tailored microstructures. 

## 4. Materials and Methods

### 4.1. Synthesis of the ST and STH MICROSCAFS^®^

The porous ST MICROSCAFS^®^ were prepared using a method derived from a previous work [3]. The first step is the silane (silica precursor) pre-hydrolysis. A total of 16.3 mL of tetraethyl orthosilicate (TEOS, 98%, Sigma-Aldrich, St. Louis, MO, USA), 13.8 mL of (GPTMS (Xiameter OFS-6040, >98.5%, Dow, Midland, MI, USA), 11.6 mL of 0.28 M HCl (aq.), and, in the case of the STH, 7.49 g of hafnium dichloride oxide octahydrate (HfOCl_2_.8H_2_O, >98%, Alfa Aesar, ThermoFisher Scientific, Waltham, MA, USA) were mixed at ambient temperature in a closed vessel under magnetic stirring for 65 min. In parallel, 10 mL of titanium (IV) isopropoxide (TiPOT, 98%, Acros Organics, ThermoFisher Scientific, Waltham, MA, USA), which is the titania precursor, and 8 mL of glacial acetic acid (≥99%, Fisher Chemical, ThermoFisher Scientific, Waltham, MA, USA) were mixed and subjected to stirring at ambient temperature for 45 min.

For the preparation of the emulsion, 114 mL of decahydronaphthalene (mixture of cis and trans isomers, 98%, Merck, Darmstadt, Germany), 45 mL of distilled water, and 6 mL of sorbitan monooleate surfactant (Span^®^ 80, HLB: 4.3, Merck, Darmstadt, Germany) were mixed at 13,000 rpm for 10 min using a homogenizer (IKA T18 digital ULTRA-TURRAX^®^, IKA, Staufen, Germany). This was added to the reactor at a mechanical stirring speed of 600 rpm and 50 °C. The reactor setup was composed of a three-neck round bottom flask reactor, a heating mantle and a thermocouple, together with a mechanical stirring device.

Subsequently, a mixture of the previously prepared titania precursor solution and the hydrolyzed silica precursors was added to the reactor, followed by the addition of 16.5 mL of ammonia in a 25% aqueous solution (Chem-Lab, Zedelgem, Belgium).

Afterwards, 1 h and 30 min later, the reactor mixture was filtrated under vacuum and washed with acetone, and the resulting MICROSCAFS^®^ were dried overnight at 45 °C. 

### 4.2. Optical Microscopy

A MSZ 5600 optical microscope (KRÜSS, Hamburg, Germany) was used to study the evolution of the emulsion and particle sizes during the different stages of the synthesis, as well as to evaluate the MICROSCAFS^®^ size, shape and maturity (stiffness qualitatively assessed by punching or tearing with the point of a needle).

### 4.3. Scanning Electron Microscopy

SEM images and EDS data were obtained using a Phenom ProX G6 benchtop SEM (ThermoScientific, Waltham, MA, USA) with the aim of assessing the dried MICROSCAFS^®^ morphology, including internal porosity. A 15 nm layer of gold–palladium was sputtered on the samples before observation using a turbomolecular pumped coater Q150T ES (Quorum Technologies, Lewes, UK).

### 4.4. Cryo-Scanning Electron Microscopy

Cryo-SEM photos and EDS data were obtained using a high-resolution scanning electron microscope with X-Ray Microanalysis and Cryo-SEM experimental facilities, namely a JSM 6301F microscope (JEOL, Ltd., Akishima, Tokyo, Japan), an INCA Energy 350 EDS spectrometer (Oxford Instruments plc, Abington, Oxfordshire, UK) and an ALTO 2500 cryo transfer system (Gantan, Pleasanton, CA, USA).

Aliquots from the reaction medium were taken at various stages of the MICROSCAFS^®^ synthesis and rapidly cooled (by inserting them into sub-cooled nitrogen), then transferred under vacuum to the cold stage of the preparation chamber. The samples were subsequently broken and coated with Au/Pd by sputtering for 45 s before being placed into the SEM chamber and analyzed at a temperature of −150 °C.

## Figures and Tables

**Table 1:** EDS atomic concentrations of the different regions of the sample by cryo-SEM.

Cryo-SEM Photomicrograph Region	Stage of the Synthesis	Atomic Concentration, %					
		C	O	Si	Cl	Ti	Hf
E1	Stage 1: Emulsion stage	92.8	7.2	-	-	-	-
E2		9.8	90.2	-	-	-	-
H1	Stage 1: Pre-hydrolysate (precursors colloidal solution, before adding to the emulsion)	35.2	46.5	14.8	-	3.5	-
H2		36.4	47.7	13.0	0.5	2.4	-
EH1	Stage 2: After addition of the pre-hydrolysate to the emulsion (embryonic stage)	24.4	43.1	24.1	-	8.4	-
EH2		26.7	48.2	21.6	-	3.5	-
EH3		29.7	65.6	2.9	1.1	0.2	0.5
EH4		96.4	3.6	-	-	-	-
EH5		29.8	60.1	6.4	1.4	1.5	0.8
EH6		19.8	73.4	5.1	-	0.9	0.8
G1	Stage 3: After addition of the ammonia solution to catalyze the gelation (final stage)	21.5	72.3	4.7	-	1.5	-
G2		94.8	5.2	-	-	-	-
G3		24.5	67.6	5	1.4	1.1	0.4
G4		92.8	7.2	-	-	-	-

## Data Availability

No new data were created or analyzed in this study. Data sharing is not applicable to this article.
